# Potential Diagnostic Properties of Chest Ultrasound in Thoracic Tuberculosis—A Systematic Review

**DOI:** 10.3390/ijerph15102235

**Published:** 2018-10-12

**Authors:** Francesco Di Gennaro, Luigi Pisani, Nicola Veronese, Damiano Pizzol, Valeria Lippolis, Annalisa Saracino, Laura Monno, Michaëla A.M. Huson, Roberto Copetti, Giovanni Putoto, Marcus J. Schultz

**Affiliations:** 1 Department of Infectious Diseases, University of Bari “Aldo Moro”, 70121 Bari, Italy; cicciodigennaro@yahoo.it (F.D.G.); annalisa.saracino@gmail.com (A.S.); laura.monno@uniba.it (L.M.); 2 Doctors with Africa, 35128 Padova, Italy; g.putoto@cuamm.org; 3 Mahidol Oxford Tropical Medicine Research Unit, Bangkok 10400, Thailand; luigipisani@gmail.com (L.P.); marcus.j.schultz@gmail.com (M.J.S.); 4 Department of Intensive Care; Academic Medical Center, 1005 AZ Amsterdam, The Netherlands; valerie.lippolis@gmail.com; 5 Department of Medicine (DIMED)-Geriatrics Section; University of Padova, 35128 Padova, Italy; ilmannato@gmail.com; 6 Doctors with Africa, Research Unit Beira, Beira 1316, Mozambique; 7 Department of Internal Medicine, Academic Medical Center, 1011 Amsterdam, The Netherlands; m.a.huson@amc.uva.nl; 8 Department of Emergency Medicine; Latisana Hospital, 33053 Latisana, Italy; robcopet@gmail.com

**Keywords:** tuberculosis, lung ultrasound, low-resource settings

## Abstract

*Background*: Chest ultrasound (CUS) has been shown to be a sensitive and specific imaging modality for pneumothorax, pneumonia, and pleural effusions. However, the role of chest ultrasound in the diagnosis of thoracic tuberculosis (TB) is uncertain. We performed a systematic search in the medical literature to better define the potential role and value of chest ultrasound in diagnosing thoracic tuberculosis. *Aim*: To describe existing literature with regard to the diagnostic value of chest ultrasound in thoracic tuberculosis. *Methods*: MEDLINE, EMBASE, and Scopus databases were searched for relevant articles. We included studies that used chest ultrasound for the diagnosis or management of any form of thoracic tuberculosis, including pulmonary, pleural, mediastinal, and military forms. *Results*: We identified five main fields of chest ultrasound application: (1) Detection, characterization, and quantification of TB; (2) detection of residual pleural thickening after evacuation; (3) chest ultrasound-guided needle biopsy; (4) identification of pathologic mediastinal lymph nodes in children; and (5) identification of parenchymal ultrasound patterns. Effusion was also detected, in early stages, with signs of organization in 24–100% of patients. A low to moderate (10–23%), false negative rate was reported for chest ultrasound-guided needle biopsy. CUS was able to identify mediastinal lymph nodes in as many as 67% of patients with negative chest radiography. *Conclusions*: Very few studies with important methodological limitations analyze the role of chest ultrasound in the diagnosis of TB. The scarce available data suggests potential targets of future diagnostic or feasibility trials, such as the detection of tuberculosis–related pleural effusion, residual pleural thickening, lymphadenopathy, TB parenchymal patterns, or the use of CUS in biopsy guidance.

## 1. Introduction

In 2015, an estimated 10.4 million patients were newly diagnosed with tuberculosis (TB) worldwide, with 1.4 million cases resulting in death [1]. To achieve the End TB 2035 target [1] of a 90% reduction in the TB incidence rate by 2035, an acceleration of the present decline rate is urgently needed. Early and accurate diagnosis in high- and low-resource settings is the first pillar of the End TB strategy and is pivotal in enabling early treatment [2].

One of the main reasons for the under-detection of TB is the limited availability of diagnostic methods [3]. Moreover, when a diagnostic apparatus is available, many patients present with non-specific symptoms and negative laboratory exams. This delays diagnosis and aggravates prognosis.

The diagnosis of active TB has always used a composite approach, uniting radiology with three other technologies: Microscopy (sputum smears), culture-based methods, and molecular tests [4]. Unfortunately, facilities using these technologies are not always available in resource-limited settings, especially in peripheral health centers.

Point-of-care chest ultrasonography (CUS), intended for lung, pleural, and mediastinal ultrasonography, is becoming an attractive, non-invasive medical imaging modality in both affluent and resource-limited settings [5]. A recent consensus standardized terminology and indications, regarding critical care lung ultrasound [6]—a method which evolved as a highly sensitive and specific imaging tool for diagnosing chest conditions, such as pneumothorax, pneumonia, and pulmonary edema. Resource-limited settings are of special interest, as radiological equipment and expertise are scarce, or even absent, due to their high costs or poor maintenance. A focused assessment of extra-pulmonary TB has been also proposed [7]. However, the role of lung, and more broadly, chest ultrasound in defining TB has been scarcely investigated. It is currently unclear if CUS could play a clinically-relevant role in diagnosing or excluding active tubercular chest lesions.

The aim of this review is to describe existing studies that have used CUS for the diagnosis of thoracic TB, to ascertain whether it represents a useful tool for the diagnosis and follow up of TB.

## 2. Methods

This systematic review was conducted, following the Preferred Reporting Items for Systematic reviews and Meta-Analyses (PRISMA) [8] statement. The methodological quality of each study was critically evaluated, using the Quality Assessment of Diagnostic Accuracy Studies-2 (QUADAS 2) [9].

### 2.1. Search Strategy

Two investigators (FG and LP), independently and unaware of the other investigator’s decisions, searched PubMed, EMBASE, and Scopus for studies assessing the role of lung ultrasound in thoracic TB. Searches were carried out without language restrictions and from the database’s inception until April 2017. Any inconsistencies that remained after discussion were resolved by consensus with a third author (NV), available for mediation. In PubMed, we used the following search strategy: (tuberculosis) AND (ultrasound OR ultrasonography) OR (miliary OR lung OR chest) AND/OR (recovery OR pattern OR diagnosis OR effusion). A similar search strategy was run in EMBASE and Scopus. Reference lists of included articles and those relevant to the topic were hand-searched for identification of additional, potentially-relevant articles.

### 2.2. Study Selection

We included all studies reporting patient data (including original studies, case-reports, and case series) using chest ultrasound for the diagnosis or management of any form of thoracic tuberculosis, namely its pulmonary, pleural, mediastinal, and miliary forms. Studies were excluded if they (1) used ultrasound for non-thoracic organs or (2) used endoscopic ultrasound. Studies were included, irrespective of the methodology employed. This was to ensure that a comprehensive understanding of the available evidence was achieved.

### 2.3. Data Extraction and Analysis

The two authors (FG, LP) independently extracted data, using a standardized spreadsheet. Any disagreement was resolved by consensus with a third author (DP). The following information was extracted: (i) Study population characteristics (i.e., author, year, number of participants, mean age, and percentage of females); (ii) study design and inclusion criteria; (iii) details of the ultrasound procedure; (iv) diagnostic criteria used for detecting TB and the final diagnosis made (pulmonary, pleural, or mediastinal TB); and (v) the anatomical site of investigation (pleural cavity, mediastinum, or lung).

The extracted information was organized in tables, describing each study’s characteristics, methods, and its main results. Patient characteristics—such as age, number of participants, and number of female cases—were summarized as the mean or proportions, where appropriate. Due to the different design of the studies included, the purely descriptive aim of 8 of the 12 studies, and the high heterogeneity of the outcomes reported, we decided not to meta-analyze the selected studies, instead reporting the main findings as descriptive results. Where some degree of diagnostic accuracy was reported, we calculated it as the percentage of patients with a positive ultrasound (US) examination, over the total of confirmed or suspected TB patients.

## 3. Results

**Figure 1:**
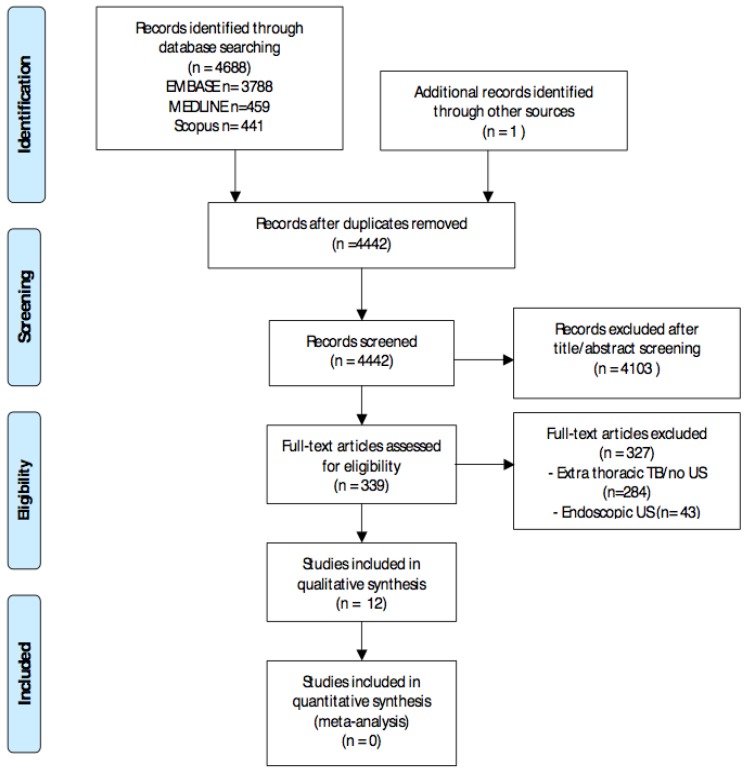
PRISMA (Preferred Reporting Items for Systematic Reviews and Meta-Analyses) flow diagram. Abbreviations: Tuberculosis (TB) and ultrasound (US).

The search identified 4442 non-duplicate records. After title and abstract screening, as well as a full text review, 12 studies were included, as shown in Figure 1 [10,11,12,13,14,15,16,17,18,19,20], with one study included from the reference screening of a previous article [21].

### 3.1. Study and Patient Characteristics

Study and patient characteristics are summarized in Table 1. The 12 analyzed studies included 300 participants, with a mean age of 32, 46% of which were females. We found that four studies were conducted in Asia, five in Europe, and three in Africa. Hemi-thoraxes (costophrenic recesses) were the most common site analyzed (*n* = 5), followed by the mediastinum (*n* = 4) and lung parenchyma (*n* = 3). Reference methods for the confirmation of tuberculosis strongly differed across studies.

### 3.2. Main Findings

The main findings of the included studies are reported in Table 2. We found that five main fields of application of chest ultrasound in the field of thoracic TB emerged. The first concerned the diagnosis of pleural effusions, initially investigated in 1989, in 21 young subjects [10]. Effusions seemed to facilitate the determination of underlying lung lesions [12], while CUS could detect pleural effusions in early stages [16]. Secondly, CUS seemed to be accurate in detecting residual pleural thickening after evacuation [20].

A third use of chest ultrasound focused on biopsies of TB lesions, as reported in three studies. In two studies, some degree of accuracy could be extrapolated, which resulted in moderate to high (76–90%) rates of success [13,21].

The fourth application aimed at identifying pathological mediastinal lymph nodes in children with suspected mediastinal disease, as reported by three studies, all in the pediatric setting [14,15,21] with variable diagnostic performance (40–100%). Only one study monitored the response after treatment, using the reduction of lymph node volume as an indicator for treatment success [14].

Finally, CUS was used to describe parenchymal patterns in one study, which only enrolled patients with miliary TB [18] and reported on only one case [19].

The qualitative assessment of the included studies showed a predominance of high or unclear risk of bias and concern over applicability, across all features analyzed, especially with regards patient selection and the reference standard used.

## 4. Discussion

The main finding of our systematic review is that the few available studies on chest ultrasound have focused on five fields of interest: detection of pleural effusion, assessment of residual pleural thickening, the helpfulness of trans-thoracic needle biopsy, assessment of mediastinal lymphadenopathies, and detection of pulmonary involvement in miliary TB. To our knowledge, this is the first review summarizing evidence for the use of chest ultrasound in the diagnosis of thoracic tuberculosis.

Surprisingly, apart from a single reported case, we found no study analyzing parenchymal ultrasound patterns, typical of pulmonary TB. Considering the pulmonary involvement of TB has been extensively studied by other radiological means [22], we expected to find evidence to include or exclude CUS as an alternative imaging technique in active TB. In the wider field of chest ultrasound, lung ultrasound (LUS) has gained acceptance for the diagnosis of consolidations and interstitial syndromes [23], hence its potential role in defining TB-related, pneumonic infiltrates. However, there appears to be scant available data at the moment, analyzing the use of LUS in the definition of TB parenchymal infiltrates.

The only study that attempted to report lung patterns describes the presence of B-lines and subpleural granularity, but only enrolled ten patients with miliary TB, which is distinct from pulmonary TB. Additionally, B-lines are, by definition, a highly non-specific sign of interstitial involvement, hence their role must be confirmed before LUS can be used to differentiate TB from other interstitial and alveolar lung diseases. Further, the presence, severity, and distribution of subpleural consolidation in patients with sole pulmonary involvement is still unknown.

There is an increasing interest in employing chest ultrasound in low- and middle-income countries [24]. Chest ultrasound has a relatively steep learning curve: It is ionization-free and is increasingly available [25] at reasonable costs. Moreover, it can be portable and operated with rechargeable batteries. Ultrasound gel, the only routine supply item needed, can easily be produced locally [26], thus making it an attractive option in resource-limited settings. On the other hand, inter-observer variability and diagnostic errors represent important pitfalls, and should be investigated specifically for TB. While the conditions for a wider implementation are favorable, none of the studies were performed in low-income countries. This observation, added to the paucity of available evidence, indicates that the use of CUS for the diagnosis of thoracic TB is still a clinical niche.

No conclusions can be drawn on diagnostic accuracy, as we did not find any clinical trial that compared CUS versus other imaging modalities. In the search for early diagnosis techniques, high negative predictive values become an important endpoint to target. If the benefits of point of care ultrasonography can be transposed to the diagnosis and management of TB—a disease that may yield highly unspecific signs [12]—it remains yet to be challenged by an adequately-powered, diagnostic trial.

The strength of our review stems from the strict search performed and pragmatic clinical questioning. Limitations of the review are the paucity of available data and extreme heterogeneity of the studies included, in terms of aim, methodology, and outcomes. The low or unclear quality of many of the included studies also greatly limits generalizations. Additionally, only five studies [12,13,14,15,21] compared the result of the CUS technique to a reference standard for the diagnosis of TB. Most importantly, no prospective studies comparing CUS to other imaging modalities in diagnosing TB were identified.

## 5. Conclusions

There is a striking scarcity of studies analyzing the use of chest ultrasound as an imaging technique to aid TB diagnosis. CUS is a promising imaging technique for the detection of TB-related effusion, residual pleural thickening, mediastinal lymphadenopathy, and trans-thoracic biopsy guidance. CUS may prove beneficial, especially in low-income countries, where incidence is high and radiological techniques are scarce. Further research is urgently needed to define pulmonary TB patterns, the feasibility of CUS, and diagnostic accuracy.

## Figures and Tables

**Table 1:** Descriptive characteristics of the study participants, study design, and chest ultrasound (CUS) techniques used.

Author, Year	Country	Study Design	N of Participants	Mean Age	Percentage of Females	Diagnostic Criteria for TBC	Final Diagnosis Made	Details of Ultrasound Procedure	Site of Investigation
Martinez, 1989 [10]	Spain	Case series	21	22.5	57	Microbiological	TB pleurisy	identification of effusion	Hemi-thoraxes
Akhan, 1992 [11]	Turkey	Case series	20	30.5	35	Pleural fluid analysis positive for MT	TB pleurisy	identification of effusion	Hemi-thoraxes and pleural line
Yuan, 1993 [12]	China	Case series	13	53.5	NA	Culture of MT	Pulmonary TB	percutaneous US-guided biopsy	Lung lesions
Gulati, 2000 [13]	India	Case series	26	35.0	31	Composite^§^	TB-lymphoadenopathy	percutaneous US-guided FNAB	Mediastinum
Bosch-Marcet, 2004 [21]	Spain	Retrospective	32	6	47	Chest radiography/CT	TB-lymphoadenopathy	identification of lymphoadenopathy	Mediastinum
Bosch-Marcet, 2007 [14]	Spain	Retrospective	57	6	NA	Chest radiography	TB-lymphoadenopathy	identification of lymphoadenopathy	Mediastinum
Moseme, 2014 [15]	South Africa	Pilot study	30	6	NA	Not specified	TB-lymphoadenopathy	identification of lymphoadenopathy	Mediastinum
Ahuja, 2014 [16]	India	Case report	1	27	100	Culture of MT	TB pleurisy	identification of effusion	Hemi-thoraxes
Bahr, 2014 [17]	Egypt	Case series	2	NA	NA	Biopsy positive for TB	Pulmonary and TB pleurisy	percutaneous US-guided biopsy	Hemi-thoraxes and Lung lesions
Hunter, 2016 [18]	South Africa	Case series	10	33	50	Sputum positive	Pulmonary TB	Lung US	Lung
Heuvelings, 2016 [19]	Netherlands	Case report	1	44	0	Chest radiography/ Bal positive for MT	Pulmonary TB	Lung US	Lung
Lai, 2016 [20]	China	Prospective study	87	64.2	32	Pleural fluid analysis positive for MT	TB pleurisy	identification of pleural effusion and RPT	Hemi-thoraxes

Abbreviations: Mycobacterium tuberculosis (MT), ultrasonography (US), tuberculosis (TB), fine-needle aspiration biopsy (FNAB), bronchoalveolar lavage (BAL), residual pleural thickening (RPT), and not available (NA). §Combination of clinical, imaging, and laboratory parameters + FNAB result.

**Table 2:** Main findings of the studies included.

Author, Year	Sensitivity of US Procedure *	Main Findings
Martinez, 1989 [10]	Not applicable	First study to propose ultrasound as a useful method for characterizing TB-related pleural effusion. US revealed winding or filiform structures in all patients associated with exudates having high fibrin and protein content.
Akhan, 1992 [11]	Not applicable	90% of patients with TB-related effusion demonstrated regular pleural thickening (1–13 mm). A total of 30% had pleural nodules and 90% of the effusions presented delicate, complete septations.
Yuan, 1993 [12]	90%	US-guided aspiration biopsy provided the diagnosis in 9 out of 10 patients. US findings of pulmonary TB with unusual radiographic appearances were even more diverse than the plain radiographs had depicted. While the US findings of hypo-, iso-, or hyperechoic consolidations and fluid bronchograms were not specific for pulmonary tuberculosis, US indicated the most appropriate area for aspiration.
Gulati, 2000 [13]	76%	A total of 20 out of 26 patients with mediastinal tuberculosis could be diagnosed by guided FNAB. No procedure-related complications were recorded. US-guided FNAB was falsely negative in 6 patients.
Bosch-Marcet, 2004 [21]	90.5%	US showed lymphoadenopathy in 67% of children with a normal chest radiograph and in 90.5% of children with chest radiographic images, compatible with TB.
Bosch-Marcet, 2007 [14]	Not applicable	US examination detected mediastinal lymphadenopathy in all children and a reduction in volume, after 3 months of treatment in 80.9% of children. Mediastinal sonography appears to have been a valuable tool for the diagnosis of TB and in the monitoring of response to treatment, in children.
Moseme, 2014 [15]	40%	Pilot feasibility study showing how mediastinal sonography could detect mediastinal lymphadenopathy in 12 out of 30 children with suspected primary TB. Care was warranted with the deeper aorto-pulmonary zones, as they were harder to visualize.
Ahuja, 2014 [16]	Not applicable	A bedside lung US was the key factor in the early diagnosis of pleural effusion and subsequent patient management.
Bahr, 2014 [17]	Not applicable	In patients with undiagnosed pleural effusion, US-guided pleural biopsies aided in the differential diagnosis of 80% of cases, indicating a tuberculous effusion in 2 out of 30 patients (6.7%).
Hunter, 2016 [18]	Not applicable	Interstitial pattern with B-lines disseminated in multiple lung areas and a pattern of bilateral, diffuse sub-pleural granularity as typical changes seen in lungs of all patients with miliary TB.
Heuvelings, 2016 [19]	Not applicable	Lung ultrasound on a single patient revealed a subpleural consolidation with “shred signs,” compatible with infiltrative TB disease.
Lai, 2016 [20]	Not applicable	A complex, septated sonographic pattern was a useful sign to predict the development of residual pleural thickening, 1 year after the start of anti-TB treatment (PPV 84%, NPV 94%).

Abbreviations: Computer tomography (CT), ultrasound (US), positive predictive value (PPV), and negative predictive value (NPV). * was calculated as % of a positive US examination, for the chosen endpoint, over the total of confirmed or suspected TB patients. Not applicable in qualitative studies.

## Abbreviations

CUS	chest ultrasound
TB	tuberculosis
